# Investigating the Phytochemical Profile and Antioxidant Activity of Different Solvent Extracts of *Sesamum prostratum* Retz.

**DOI:** 10.3390/plants14040519

**Published:** 2025-02-08

**Authors:** Felix Irudhyaraj Dhanaraj, Jagadheesh Kumar Kalimuthu, Pavan Santhosh Balamurugan, Punitha Subramani, David R. Katerere, Manikandan Gurusamy

**Affiliations:** 1 Department of Botany, Bishop Heber College (Autonomous), Affiliated to Bharathidasan University, Tiruchirappalli 620017, Tamil Nadu, India; 2 Pharmaceutical & Biotech Advancement in Africa (PBA2), Department of Pharmaceutical Sciences, Tshwane University of Technology, Arcadia Campus, Pretoria 0001, South Africa

**Keywords:** phytochemicals, GC/MS analysis, ethanol, benzenedicarboxylic acid, DPPH

## Abstract

Numerous bioactive chemicals with a range of advantageous effects on human metabolism can be found in medicinal plants. The knowledge of these phytochemicals is essential for the identification of potential medicines. In the present study, *Sesamum prostratum* was selected for GC-MS profiling and antioxidant studies. Plant extracts were prepared with ethanol, ethyl acetate, and acetone by using the continuous hot Soxhlet extraction method. The chemical compositions were analyzed by GC/MS. In addition, antioxidant activity was measured using ABTS, DPPH, and hydrogen peroxide assay. The GC-MS analysis of the different solvent extracts showed a total of 32 compounds present in *S. prostratum*. Ethanol extracts showed 11 compounds, ethyl acetate extracts showed 8 compounds, and acetone extracts showed the presence of 13 compounds. Different dominant chemicals were identified in each solvent extract by the phytochemical study. The chemical composition of the extracts revealed notable differences. The ethanol extract was dominated by 1,2-benzenedicarboxylic acid, which comprised 51.4% of the total composition. In contrast, the acetone extract had 3-ethyl-4-methylhexane as its primary component, while the ethyl acetate extract was characterized by the presence of 1,2-benzenedicarboxylic acid as its major chemical constituent. The different assay on free radical scavenging activity of the extracts showed antioxidant activity. The acetone extracts showed the least IC_50_ value of 35.13 μg/mL for the hydrogen peroxide test, the ethyl acetate showed IC_50_ value 65.49 μg/mL in the ABTS assay, and the ethanol extract showed the lowest IC_50_ value of 80.84 μg/mL in the DPPH assay. The results indicated that the plant has bioactive compounds with antioxidant potential that can be further investigated for anticancer and other medicinal uses.

## 1. Introduction

Plants are used medicinally in different countries, and are a source of many potent and powerful drugs [1]. Medicinal plants were traditionally used by human beings from ancient times for their therapeutic value. Generally, the medicinal plants were collected by the local people for household ailments. Much knowledge on the uses of these medicinal plants has not been explored due to the lack of transfer of knowledge. The World Health Organization (WHO) reported that 80% of the world population relies on traditional medicine for primary healthcare needs [2]. Angiosperm plants are important sources of phytochemical compounds that play a major role in human healthcare maintenance [3]. Known for their antiviral, antibacterial, and antifungal qualities, medicinal plants are used in both conventional and contemporary treatments all over the world. In drug discovery, phytochemical substances originating from these plants are essential for creating analogs of pharmaceutical products [4,5,6,7,8,9,10]. Beyond human therapy, they are used in veterinary care, agriculture, and a number of scientific research domains [11].

For the identification of the phytochemical in plants, several methods were applied. Mass spectrometry (MS) is the direct examination and identification of used in medicinal plants, especially when combined with chromatographic methods such as gas chromatography–mass spectrometry (GC-MS). The high speed of determination, analysis and sensitivity to trace elements makes gas chromatography as an efficient tool for the separation of complex samples and, coupled with mass spectrometry, it helps in the direct identification of unknown chemical compounds in the sample [12].

Most of the compounds present in plants are also identified as having antioxidant properties. One of the important processes during metabolism is the formation of reactive oxygen species, which may damage cellular components [13]. Physiologically, under normal conditions, free radicals are produced and readily scavenged by endogenous antioxidants. If the formation of free radicals is higher than the amount of endogenous antioxidants, then they may cause metabolic stress. The excess free radicals may damage tissue and cause diseases such cancer, heart diseases, Alzheimer’s diseases, neurodegenerations, atherosclerosis, cataracts inflammation and aging [14]. Plant-based compounds with antioxidant properties are largely utilized to scavenge these reactive species. At present, several researchers are involved in identifying the antioxidant activities of different compounds. Phenolic phytochemicals are well known for their pharmacological properties that are effected through antioxidants. However, when combined with other phytochemicals, the biological efficacy of individual compounds may be altered or increased, highlighting the significance of synergistic interactions in plant-based medicines [15].

The genus *Sesamum* belongs to the family Pedaliaceae, which is distributed through the tropical and subtropical regions of Asia, Africa, and America. The genus consists of 36 species, of which 35 are wild species and one is cultivated (*Sesamum indicum*). Among the 35, species 19 are indigenous to Africa and India, and are considered the primary center of origin of *Sesamum*. Derived from the sesame plant, sesame oil is used extensively in Indian traditional Ayurvedic treatments, and is prized in traditional Chinese medicine for its anti-aging and revitalizing qualities [16]. According to Astalakshmi et al. [17], the genus *Sesamum* is characterized by a diverse range of chemical constituents, including flavonoids, alkaloids, essential oils, fatty acids, steroids, terpenoids, polyphenols, and glycosides. These compounds are associated with numerous health benefits, such as antioxidant, anti-inflammatory, anti-diabetic, hepatoprotective, antimicrobial, and anticonvulsant properties, highlighting the potential of *Sesamum* plants as a valuable source of medicinal agents. *Sesamum prostratum* Retz. is a prostrate perennial herb generally found in the arid regions of Indian subcontinent. Astalakshmi et al. [18] conducted a preliminary phytochemical analysis of the hydro-alcoholic extract of *Sesamum prostratum* aerial parts. This evaluation revealed a diverse range of bioactive compounds, including flavonoids, glycosides, tannins, steroids, carbohydrates, proteins, and amino acids, indicating the plant’s potential for medicinal applications. The plant has not been fully explored for phytochemical profiling and antioxidant activity. Using GC/MS analysis and antioxidant assays, this study examined the phytochemical composition and antioxidant capacity of *Sesamum prostratum* in light of the importance of the *Sesamum* genus.

## 2. Results

### 2.1. Phytochemical Profiling of Extracts of S. prostratum

The present study on the GC-MS profiling of different solvents such as ethanol, ethyl acetate, and acetone extracts of *S. prostratum* recorded with different phytochemicals, which are listed in Table 1, Table 2 and Table 3. The different peaks and ion chromatogram were obtained through GC-MS analysis. The results showed that the plants contain a total of 32 compounds belonging to different classes of chemicals such as alkaloids, alkanes, aromatic benzenes, acids, and its ester forms.

The GC-MS result on the ethanol extract of *S. prostratum* showed the presence of 11 different bioactive compounds (Table 1), of which 1,2-Benzenedicarboxylic acid showed the maximum area percentage of 51.4%. The other compounds, such as Butane, 2,2-dimethyl-, Stearic Acid, Hexanedioic acid, bis(2-ethoxyethyl) ester,1,7-Diazabicyclo[2.2.0]heptane and 1-(2-Hydroxyethoxy)tridecane, are in the range of 3–4%. Most of the compounds are found to be in ester form, and other phytochemicals are in the trace percentage.

The result of GC/MS analysis of the ethyl acetate extract showed the presence of eight compounds (Table 2), of which Octadecane showed the maximum of 19.93%,which was followed by 1,4-Benzenedicarboxylic acid, bis(2-ethylhexyl) ester with 7.48%. The other compounds, such as Hexane, 3,3-dimethyl-, 2-[2-(Benzoyloxy)ethoxy]ethyl benzoate and Cyclododecanamine, were recorded with area percentages of 3–5%.

The studies on the GC/MS chemical profile of acetone extract showed the presence of 13 compounds (Table 3), of which 1,4-Benzenedicarboxylic acid, bis(2-ethylhexyl) ester showed the maximum area percentage of 25.7%, 1,2-benzenedicarboxylic acid showed area percentage of 20.09%, and most of the other phytochemicals were recorded with 2–5%.

### 2.2. Antioxidant Assay on Different Solvent Extract of S. prostratum

#### 2.2.1. ABTS Assay on Free Radical Scavenging of *S. prostratum* Extracts

**Figure 1:**
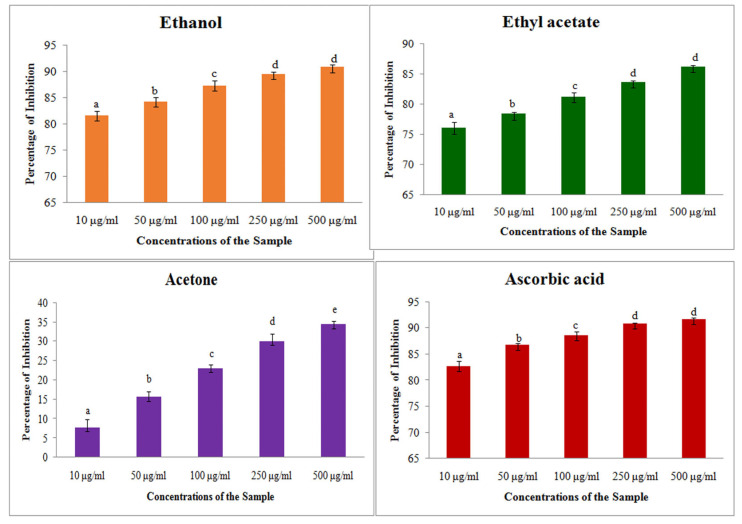
ABTS assay on different solvent extract of *S. prostratum*. Each value is the Mean ± SD of triplicates analysis; each set of bars presented on antioxidant properties—ABTS assay (a–e) on crude extract of ethanol, ethyl acetate, acetone and ascorbic acid with different superscript letters are statistically significant (one-way ANOVA test: *p* < 0.05; further, the post hoc multiple comparisons with SNK test).

The free radical scavenging activity through ABTS assay showed that the antioxidant activity was increased along with the increase in extract concentration from 50 to 500 μg/mL. It showed a maximum percentage of 53 with the ethyl acetate extract at a 500 μg/mL concentration, and the other two solvents showed maximum of 34% in ethanol extract and 34% in acetone extract. On comparing with the inhibition percentage of ascorbic acid, the plant extract showed lower activity (100 μg/mL and 250 μg/mL) (Figure 1).

The IC_50_ value showed that the ethyl acetate extract has the minimum concentration 65.5 to scavenge the free radicals than the other two extracts (Table 4). Though the IC_50_ value was higher than the standard ascorbic acid, all of the extracts showed significant antioxidant activity against the free radicals. The mean inhibition percentage of ABTS assay with different solvent extracts were clearly presented in Figure 1.

#### 2.2.2. DPPH Assay

**Figure 2:**
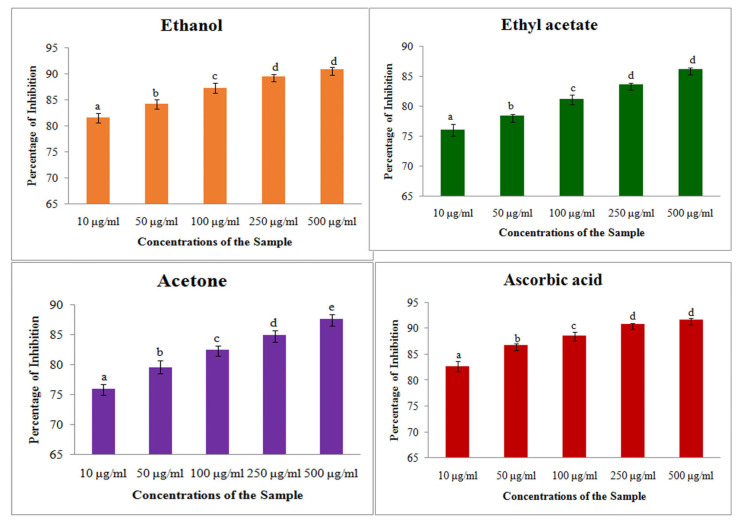
DPPH assay on different solvent extracts of *S. prostratum*. Each value is the Mean ± SD of triplicates analysis; each set of bars presented on Antioxidant—DPPH (a–e) on crude extract of ethanol, ethyl acetate, acetone and ascorbic acid with different superscript letters are statistically significant (one-way ANOVA test: *p* < 0.05, further, the post hoc multiple comparisons with SNK test).

DPPH assays on different solvent extracts showed concentrations of proportional free radical scavenging activity, where the increasing the concentration of extract showed increased antioxidant activity (Figure 2). Among the three different solvent extracts, the maximum inhibition percentage was showed by ethanol extract with 90.84 ± 0.43%, and it was slightly lower than the standard ascorbic acid 91.92 ± 0.60%. The other two extracts also showed antioxidant activity, which is slightly less than the standard inhibition percentage. The IC_50_ of three different solvents showed that ethanol extract has the least concentration of 80.84 μg/mL than the other extracts (Table 4).

#### 2.2.3. H_2_O_2_ Assay

**Figure 3:**
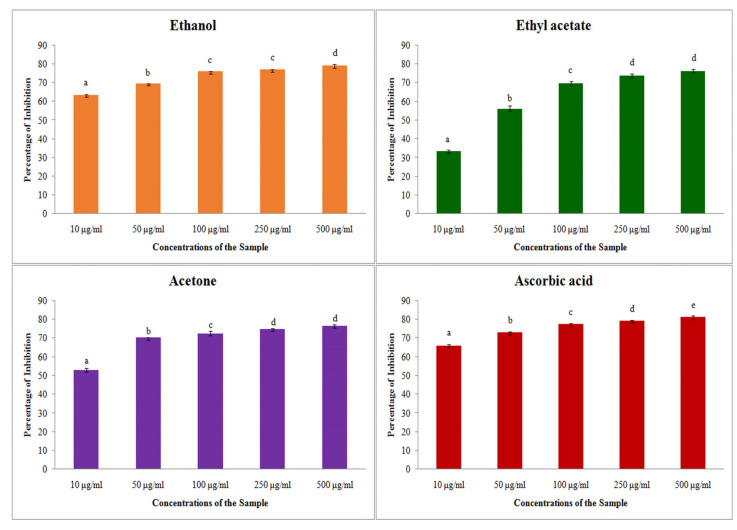
H_2_O_2_ assay on different solvent extract of *S. prostratum*. Each value is the Mean ± SD of triplicate analyses; each set of bars presented on antioxidant—hydrogen peroxide scavenging properties (a–e) on crude extract ethanol, ethyl acetate, acetone and ascorbic acid with different superscript letters are statistically significant (one-way ANOVA test: *p* < 0.05; further, the post hoc multiple comparisons with SNK test).

The antioxidant assay performed with free radical scavenging of hydrogen peroxide showed that all of the plant extracts had the ability to scavenge the free radicals. The inhibition percentage for the H_2_O_2_ was increased with the increasing concentration of the extracts (Figure 3). From the table, it was clearly noted that the inhibition percentage of extracts have slightly lesser antioxidant activity than the standard. All of the three solvents have a notable antioxidant activity against the hydrogen peroxide. The maximum inhibition percentage was obtained from ethanol extract (78.93 ± 0.81), but it was much less than the standard ascorbic acid (81.33 ± 0.80). On comparing, the inhibition activity of standard acetone showed the least difference in inhibition percentage (with difference of 2.43%). Further IC_50_ values obtained for the hydrogen peroxide assay was also very low for all of the solvent extracts, such as ethanol (58.91 μg/mL), ethyl acetate (42.45 μg/mL) and acetone (35.13 μg/mL), respectively (Table 4). The results show that the plant consists of biologically active compounds with high potentials for antioxidant activity.

## 3. Discussion

### 3.1. Phytochemical Profiling of Different Solvent Extract of S. prostratum

Phytochemicals are compounds derived from plants which maybe medicinally important for various ailments. Profiling of such compounds with the help of modern technologies benefits us in identifying and selecting plants of high potential to isolate and enumerate the biologically active compounds. GC-MS is a boon in this field, which renders enormous applications in the identification of various compounds presents in the plant extracts with minimum amounts of sample, and identifies trace amounts of different bioactive substances. It is one of the best techniques to identify the constituents of volatile matters, long chained and branched chain hydrocarbons, alkaloids, acids, steroids, esters, etc. The present results showed that the plants contain a total of 32 compounds belonging to different classes of chemicals such as alkaloids, alkanes, aromatic benzenes, acids, and their ester forms.

Among the compounds recorded in three different solvent extracts, phthalic acids and their ester forms are the dominant group of compounds present in *S. prostratum* extracts. Phthalic acid is an isomer of benzene dicarboxylic acids and other isomers, namely 1,4-benzenedicarboxylic acid (terephthalic acid), were found to be present in the plant extracts of *S. prostratum*. The detected chemicals were shown to be present in 13 different medicinal plants, including *Panax pseudoginseng* subsp. *himalaicus*, according to a literature analysis conducted using PubMed, Scifinder, and the Dictionary of Natural Products (DNP). Notably, bioactivity tests have shown that it has immunomodulatory activities as a B-cell stimulant, and anticancer characteristics against PC3, MCF, HCT-116, A549, and MIAPACA cell lines [19]. This substance works well against a number of illnesses, such as syphilis, leptospirosis, amebiasis, and Rickettsia infections [20].

Succinic acid and its derivative were also reported to be present in the ethanol and acetone extracts, which was also previously reported to be present in several plants; in particular, it was extracted from the several medicinal herbs by Wang et al. [21]. The other identified compounds were also found to be medicinally important, and were also reported in several studies. Among the three solvents used, the polar (ethanol) and mid polar (acetone) extracts showed the maximum number of phytochemicals, and the nonpolar ethyl acetate extract showed a lesser number of phytochemicals.

### 3.2. Antioxidant Assay

In recent times, there several researchers involved in identifying the plants with antioxidant potential to reduce the risk of cancer and other metabolism-related diseases. Free radicals are very reactive, are produced during the normal physiological metabolism, and are readily absorbed by the endogenous antioxidants or through exogenous antioxidants. If the level of free radicals increased more than the concentration of antioxidants, it damages the nearby tissues or interferes with other physiological functions, which may lead to diseases. The present study on the three different solvents (polar, non-polar and mid polar) extracts of *S. prostratum* showed antioxidant activity in the all the tested assays. The IC_50_ value for the all the assay showed that all the extracts have antioxidant molecules.

The current results are consistent with earlier antioxidant research by Muthaya et al. [22], who found that, at 800 μg/mL, the DPPH scavenging activities of methanol extract were 131.4%, ethanol extract was 128.0%, and ethyl acetate extract was 121.7%. An accurate measure of a natural antioxidant’s ability to donate electrons is its reducing power activity [23,24]. Plant extracts’ reducing power and antioxidant activities have been directly linked in a number of studies [23,25]. The concentration-dependent rise in the inhibition percentage of various solvent extracts in this investigation suggests that the *S. prostratum* extract’s reductive capacity is on par with that of regular ascorbic acid. Generally, the antioxidant studies were conducted on the oils obtained from the seeds of the genus *Sesamum*, with fewer studies on the plants extracts. The present study indicates that not only the oils obtained from the seeds, but also the whole plants contain antioxidant potential compounds.

## 4. Materials and Methods

### 4.1. Collection and Preparation of Plant Samples

**Figure 4:**
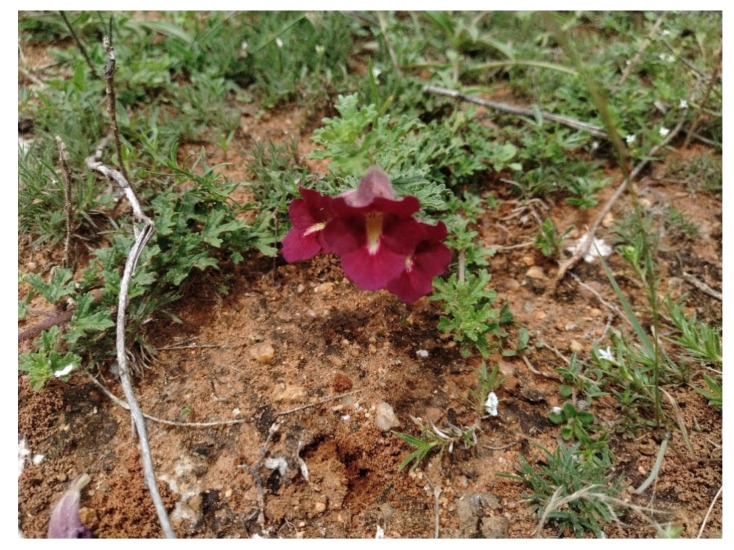
*Sesamum prostratum* Retz. habit.

The fresh plants of *Sesamum prostratum* Retz. (Figure 4) were collected from Trichy, TN, India, between June and August 2023. The collected plant species were identified by Dr. Felix Irudhyaraj, Assistant Professor at Bishop Heber College, Trichy. The identified plant specimens were subsequently deposited at the Biology Herbarium Unit, Gandhigram Rural University, Dindigul, Tamil Nadu, India, and assigned the herbarium accession number GDU0645. The healthy plants were separated and shade-dried under room temperature. Once dried plants were powdered by using an electric blender.

### 4.2. Continuous Hot Extraction Using Soxhlet Apparatus

The extraction process was performed at 80 °C for 3 h. Successive extraction of 50 g of powdered *S. prostratum* was carried out using 250 mL of each solvent in the following sequence: ethanol, ethyl acetate, and acetone [26,27,28,29]. The extraction yield percentages were 21% (ethanol), 17% (ethyl acetate) and 11% (acetone).

### 4.3. GC/MS Analysis of Different Solvent Extracts

The extracts were subjected to GC-MS analysis utilizing a QP 2020 SHIMADZU fitted with an SH-Rxi 6511 MS capillary column. At a steady flow rate of 1.20 milliliters per minute, inert helium gas was employed as the carrying gas. The injector and mass transfer line temperatures were set at 25,000 °C and 20,000 °C, respectively. The oven was set to run at 600 °C every minute, from 5000 °C to 25,000 °C. The spit less mode was used to inject a diluted 2 µL quantity of methanol extract. Peak area normalization was used to express the relative fraction of the elements of the methanol extract. The chemical components of leaf extracts were determined by computer matching of mass spectra with those of standards (NIST14.LIB. and WILEY8.LIB.) based on GC retention time on SH-Rxi 6511 MS capillary column.

### 4.4. Antioxidant Assays

#### 4.4.1. ABTS Assay

ABTS assays on plant extracts were analyzed using the method from Arnao et al. [30] with some modifications. For the present study, 7 mM of ABTS solution and 2.4 mM of potassium persulfate solution were used as the stock solution. Then, the working solution was prepared by mixing the two stock solutions in equal quantities, and allowing them to react for 14 h in a dark at room temperature. The solution was then diluted by mixing 1 mL of ABTS solution with 60 mL of methanol to obtain an absorbance of 0.706 ± 0.01 units at 734 nm using a spectrophotometer. The solution was the control standard. Fresh solutions were made for every extract concentration (10–500 μg/mL) in order to perform the ABTS tests. After combining the extracts with 1 milliliter of ABTS solution, they were incubated for seven minutes. A spectrophotometer was used to detect the absorbance at 734 nm. Each extract’s ability to scavenge ABTS was compared to that of ascorbic acid, and the percentage of inhibition was determined.


ABTS radical scavenging activity (%) = Abs control − Abs sample/Abs control

Abs control: absorbance of ABTS radical in methanol (blank).

Abs sample: absorbance of ABTS radical solution mixed with sample extract/standard.

#### 4.4.2. DPPH Assay

The antioxidant activity of *S. prostratum* extracts were studied for scavenging effects using the DPPH free radical assay [31]. The DPPH ethanolic solution (300 μL) was added to 40 μL of plant extract with different concentrations of 10–500 μg/mL. The DPPH solution was freshly prepared before the assay, and kept in the dark at 4 °C. Ninety-six percent ethanol (2.7 mL) was added to each concentration, and the entire content was shaken vigorously. The prepared mixture was allowed to stand undisturbed for 5 min to allow for completion of the reaction. Thereafter, the absorbance value was measured with a spectrophotometer at 540 nm. Different solvent as pure was used as blank to set absorbance zero. The DPPH assay was used to assess the plant extracts’ capacity to scavenge free radicals. In parallel, a blank sample comprising ethanol and DPPH solution was made. Every test was run in triplicate.

Percent (%) inhibition of DPPH
activity = [(A − B)/A] × 100

where B and A are the absorbance values of the test and of the blank sample, respectively.

#### 4.4.3. Hydrogen Peroxide (H_2_O_2_) Assay

Hydrogen peroxide is one of the best known components used to study free radical scavenging activity. A solution comprising a 20 mM concentration of hydrogen peroxide was prepared in phosphate-buffered saline at pH 7.4. Various concentrations of plant extract (10–500 µg/mL) and standard ascorbic acid in 1 mL of ethanol were added to 2 mL of hydrogen peroxide in phosphate buffer. The mixture was left free for 10 min, and then the absorbance was measured at 230 nm [32].

% inhibition = [(Ac − As)/Ac]×100

where Ac is the absorbance of the control and As is the absorbance in the presence of the sample of *S. prostratum* extracts or standard.

#### 4.4.4. Statistical Analyses

All experiments were performed in triplicate and the results are expressed as Mean ± Standard Deviation (SD). Statistical analyses were conducted using a one-way ANOVA test: *p* < 0.05; further, the post hoc multiple comparisons with SNK test were used to determine the significance of the data.

## 5. Conclusions

Plants are a treasure trove of several phytochemicals that are medicinally important with several biological activities. Identification and quantification of such phytochemicals are essential, as they are safe, less expensive, and efficient applications without any side effects. In the present study, the phytochemical of *S. prostratum* was profiled using GC-MS and examined for the antioxidant potential. The phytochemical analysis revealed that ethanol extracts contained 11 bioactive compounds, ethyl acetate extracts had 8, and acetone extracts had 13, indicating that the phytochemicals are predominantly polar in nature. Among the compounds identified, benzenedicarboxylic acid and its esters are in higher concentration. The antioxidant studies with different assays showed that all the three solvent extracts have potential antioxidant activity with increasing concentration. This implies that the plant possesses potential antioxidant compounds. Hence, the plant can be further utilized for the isolation and quantification of bioactive compounds for medicinal purposes.

## Figures and Tables

**Table 1:** List of compounds present in ethanol extract of *S. prostratum*.

S. No.	Compound Name	MF	MW	RT	Area %	SI
1.	1-(2-Hydroxyethoxy)tridecane	C_17_H_36_O_2_	244	18.238	2.33	90
2.	Butane, 2,2-dimethyl-	C_6_H_14_	86	16.128	4.66	89
3.	1,2-Benzenedicarboxylic acid	C_24_H_38_O_4_	390	34.883	51.44	89
4.	Stearic Acid	C_18_H_36_O_2_	284	26.627	3.81	82
5.	16-Hydroxyhexadecanoic acid	C_16_H_32_O_3_	272	34.498	1.68	69
6.	1,7-Diazabicyclo[2.2.0]heptane	C_5_H_10_N_2_	98	38.355	3.33	63
7.	2-Oxo-6-(piperidine-1-sulfonyl)-benzooxazole-3- carboxylic acid cyclohexylamide	C_19_H_25_N_3_O_5_S	407	39.935	0.63	60
8.	Hexanedioic acid, bis(2-ethoxyethyl) ester	C_14_H_26_O_6_	290	38.299	3.02	58
9.	Amyl nitrite	C_5_H_11_NO_2_	117	38.675	0.53	58
10.	3-Methylbutyl hexadecanoate	C_21_H_42_O_2_	326	38.755	1.06	55
11.	3,7-Bis[(trimethylsilyl)oxy]cholest-5-ene, (3.beta.,7.beta.)-	C_33_H_62_O_2_Si_2_	546	39.68	0.85	52

MF—Molecular Formula;MW—Molecular Weight; RT—Retention Time; SI—Soft Ionization Value.

**Table 2:** List of compounds present in ethyl acetate extract of *S. prostratum*.

S. No.	Compound Name	MF	MW	RT	Area %	SI
1.	2,4-Dipropyl-5,5-dimethyl-1,3-dioxane	C_12_H_24_O_2_	200	30.123	0.58	94
2.	Octadecane	C_18_H_38_	254	16.128	19.93	93
3.	Hexane, 3,3-dimethyl-	C_8_H_18_	114	22.132	3.85	89
4.	1-(2-Hydroxyethoxy)tridecane	C_15_H_32_O_2_	244	23.935	1.7	89
5.	2-[2-(Benzoyloxy)ethoxy]ethyl benzoate	C_18_H_18_O_5_	314	34.167	3.01	89
6.	1,4-Benzenedicarboxylic acid, bis(2-ethylhexyl) ester	C_24_H_38_O_4_	390	37.5	7.48	89
7.	Cyclododecanamine	C_12_H_25_N	183	38.131	3.19	54
8.	Octadecanoicacid, 2-hydroxy-1,3-propanediyl ester	C_39_H_76_O_5_	624	37.88	1.28	47

MF—Molecular Formula;MW—Molecular Weight; RT—Retention Time; SI—Soft Ionization Value.

**Table 3:** List of compound present in acetone extract of *S. prostratum*.

S. No.	Compound Name	MF	MW	RT	Area %	SI
1.	1,4-Benzenedicarboxylic acid, bis(2-ethylhexyl) ester	C_24_H_38_O_4_	390	37.501	25.7	91
2.	1,2-benzenedicarboxylic acid	C_24_H_38_O_4_	390	34.881	20.09	88
3.	Nitrocyclopentane	C_5_H_9_NO_2_	115	24.56	0.99	83
4.	Decanoic acid	C_10_H_20_O_2_	172	26.627	2.63	83
5.	Hexane, 3-ethyl-4-methyl-	C_9_H_20_	128	39.587	4.23	77
6.	2-Tetradecynal, 4-hydroxy-	C_14_H_24_O_2_	224	29.402	2.52	76
7.	Cyclohexanone, 4-(1,1-dimethylpropyl)-	C_11_H_20_O	168	34.634	1.67	75
8.	Tetrahydropalmatine	C_21_H_25_NO_4_	355	37.585	1.44	56
9.	Furan, tetrahydro-2,5-dimethyl-	C_6_H_12_O	100	37.662	2.73	54
10.	Succinic acid, 2,3-dichlorophenyl 2,2,3,4,4,4-hexafluorobutyl Ester	C_14_H_10_Cl_2_O_4_	426	38.174	0.91	50
11.	1,2-Dimethoxy dodecane	C_14_H_30_O_2_	230	38.13	1.55	49
12.	(3S)-3-[(1′R)-1′-hydroxy-3′-methylbutyl]-8-Methoxy-3,4-dihydroisocumarin	C_15_H_20_O_4_	264	37.97	3.2	48
13.	Ethyl 5-(1-piperidinylacetyl)-10,11-dihydro-5H- dibenzo[B,F]azepin-3-ylcarbamate	C_24_H_29_N_3_O_3_	407	38.045	2.49	47

MF—Molecular Formula;MW—Molecular Weight; RT—Retention Time; SI—Soft Ionization Value.

**Table 4:** IC_50_ Value of various extract *S. prostratum* for different free assays.

Free Radical Scavenging Assay	IC_50_ Value		
	Ethanol Extract	Ethyl Acetate Extract	Acetone Extract
ABTS	67.5 μg/mL	65.5 μg/mL	82.2 μg/mL
DPPH	80.8 μg/mL	102.8 μg/mL	88.3 μg/mL
H_2_O_2_	58.9 μg/mL	42.5 μg/mL	35.1 μg/mL

## Data Availability

Data are contained within the article.
